# Gender-Specific Differences in the Relationship between Autobiographical Memory and Intertemporal Choice in Older Adults

**DOI:** 10.1371/journal.pone.0137061

**Published:** 2015-09-03

**Authors:** Maayke Seinstra, Katharina Grzymek, Tobias Kalenscher

**Affiliations:** Department of Comparative Psychology, Institute of Experimental Psychology, Heinrich-Heine University Düsseldorf, Düsseldorf, Germany; CNR, ITALY

## Abstract

As the population of older adults grows, their economic choices will have increasing impact on society. Research on the effects of aging on intertemporal decisions shows inconsistent, often opposing results, indicating that yet unexplored factors might play an essential role in guiding one's choices. Recent studies suggest that episodic future thinking, which is based on the same neural network involved in episodic memory functions, leads to reductions in discounting of future rewards. As episodic memory functioning declines with normal aging, but to greatly variable degrees, individual differences in delay discounting might be due to individual differences in the vitality of this memory system in older adults. We investigated this hypothesis, using a sample of healthy older adults who completed an intertemporal choice task as well as two episodic memory tasks. We found no clear evidence for a relationship between episodic memory performance and delay discounting in older adults. However, when additionally considering gender differences, we found an interaction effect of gender and autobiographical memory on delay discounting: while men with higher memory scores showed less delay discounting, women with higher memory scores tended to discount the future more. We speculate that this gender effect might stem from the gender-specific use of different modal representation formats (i.e. temporal or visual) during assessment of intertemporal choice options.

## Introduction

You are retired. Would you now finally spend your money on small pleasures right now, or rather save for that new car you always dreamed of having? Throughout our lives we make countless choices of this kind where the outcomes become available over time, for example when we decide to refrain from eating the tasty hamburger to go for the healthy salad instead. As these intertemporal decisions can have far reaching consequences, it is important to understand how our preferences develop over time and which factors influence our choice behavior.

Intertemporal decision making is usually assessed using monetary incentives available at specific points in the future. Because a delay has a negative effect on the subjective value of a reward, amounts available in the future are worth less (i.e. are discounted) compared to when they are received now. It is commonly found that the value of a monetary amount (or other goods) is discounted in a hyperbolic fashion [1, 2], with initial steep discounting of reward values with short delays to reward consumption, and flatter discounting with longer delays. The initial steep decline in the discount function is often related to the characteristic ‘present-bias’ which relates to the tendency to reverse preferences in favor of immediate gratification at the expense of meeting long-term goals. The general steepness of the discount function can be used as an index of subjective ‘(im)patience’, i.e., the general sensitivity to delays.

Our time preferences change with age [3–7]. Studies on the effects of age on discounting often find increased discounting in children and adolescents, with decreasing discount rates in adulthood [8–11]. The change in discounting across childhood and adolescence presumably reflects the maturation process of prefrontal areas important for executive control. With regard to older aged groups, evidence is inconsistent: while some studies found no difference in discount rates between older and younger adults [10, 12, 13], other studies suggest increasing discount rates with age [14, 15], yet others found decreasing discount rates [8, 9, 11, 16, 17]. Moreover, the findings seem to depend on the type of reward (primary or secondary) as well [16]. For very old adults, it can be argued that the shortened life expectancy renders the preference for long term outcomes more risky because they may not live to experience the realization of the future outcome [18]. On the other hand, a lifetime of decision making might increase the ability to delay gratification because the older adults have learned the value of patience through experience [8, 17, 19, 20].

However, a third possibility to explain the inconsistency in the results on old age and discounting is the great variability in age-related decline of decision-relevant mental and neural functionality: older age comes with neuronal changes in areas involved in reward processing and decision making [6, 21–26, 27, 28]. It is therefore possible that changes in discounting in older individuals depend on the (variable) degree in cognitive decline associated with older age [29]. One mental function that declines with age is episodic thinking [30, 31]. Interestingly, several studies have linked episodic future thinking, i.e., mental time travel, or imagining possible future outcomes, to delay discounting behavior [32–35]. The core finding of these studies is that episodic future thinking goes along with decreased delay discounting [33–35], supporting the hypothesis that the better one is able to imagine the future outcome, the higher the subjective valuation of that future outcome will be.

These and other studies indicated that imagining future events activates the same core neural network that is involved in episodic memory functioning [36, 37], supporting the theory that the episodic memory system is used to create images of future events in the mind’s eye [38–41]. It has been suggested that to assess whether a delayed reward is the most preferable option, one needs to have a representation of future states of oneself to determine how valuable that reward will be, and use this representation to maintain motivation for overcoming short-term temptations [35, 42, 43]. This assessment could depend on recalling similar rewards obtained in the past.

Thus, because episodic future simulation and episodic memory draw on similar neural systems, and because delay discounting might depend on recalling rewards from the past, it is tempting to speculate that episodic memory performance affects discounting behavior. However, behavioral studies linking delay discounting with episodic simulation focused on the episodic projection of *future*, not the recall of *past* events. In addition, memory recall processes that bias decisions do not necessarily need to be conscious or effortful [44]. It is therefore unclear whether more general memory processes or episodic memory mechanisms in particular play a significant role in determining discounting levels in situations where episodic future thinking is not explicitly prompted in the choice task.

Not only future rewarding events, but also immediate rewards could trigger memory processes that do not necessarily have to be episodic. High integrity of the memory network, including the hippocampus, would in that case not necessarily lead to a decrease in discounting. On the other hand, unconscious preferences for more profitable delayed rewards, potentially due to a general bias towards long-term thinking in our society, could render (unconscious) memory retrieval mechanisms an important factor for biasing choice towards delayed rewards. This would be in line with findings of Kwan et al. [45], who found that in persons with hippocampal amnesia, future-orientated decision making was relatively intact, whereas they were unable to imagine detailed future events. A more recent study found that, even though persons with hippocampal amnesia show similar discounting as healthy controls, when prompted to imagine spending future rewards amnesic patients did not show decreased discounting, whereas healthy controls did [46]. This is in line with the idea that episodic future thinking is one of the factors that influences intertemporal decision making, but shows that engagement of the hippocampal network is not a necessary requirement for discounting.

The current study could shed more light on whether episodic memory retrieval processes influence intertemporal decision making without explicitly triggering future simulation. Evidence for the importance of episodic memory, i.e. the storage and retrieval of *past* episodes, for delay discounting is elusive. In order to test this idea, it would be desirable to have a population sample with a substantial degree of variability in episodic memory performance. As mentioned, this is the case in older subjects: several studies have shown that episodic memory functioning declines with age [30, 31], but the extent of decline differs strongly between individuals [47, 48]. We therefore aimed to investigate the relationship between episodic memory functioning and discount behavior in older aged individuals. We hypothesize that age-related variability in episodic memory performance may be an important mediating variable on discounting behavior that could explain some of the discrepancies found in the literature.

Our study was designed to investigate the relationship between episodic memory performance and delay discounting in a group of older adults. We expected that episodic memory performance correlated with decreased discounting when memory performance was higher. Overall, our results do not support our hypothesis. However, we additionally explored the role of gender, as several studies have shown gender effects in episodic memory tasks [49–51, 52] and found an interaction effects of gender and autobiographical memory on discounting.

## Methods

### Participants

Sixty-two older adults (33 female) between 60 and 89 years (M = 72.60, SD = 6.47) were recruited from an internal database of the Institute of Experimental Psychology at Heinrich-Heine-University Düsseldorf. Of this sample, all participants denied to suffer from any neurological or psychiatric disease or to have been taking psychiatric medication at any time in their life. None of the participants used drugs or exceeded the limits of low-risk alcohol use (> 20 g alcohol per day for women; > 40 g alcohol per day for men). Of the three smokers in the sample, all smoked less than 20 cigarettes a day. All participants were German native speakers and scored at least 25 points (M = 28.37, SD = 1.54) in the Mini-Mental State Examination (MMSE, [53]). With the exception of two, all participants were retired.

Results of four participants were excluded from analyses due to incorrectly answering catch trials in the intertemporal choice task (see below). The results reported below are therefore based on a sample of 58 adults (30 female) between 60 and 89 years (M = 72.57, SD = 6.39). See Table 1 for further demographic information. Participants received a general allowance of 5 Euro immediately after participation. Additionally, participants were paid according to their choice in the intertemporal choice task between 10 Euro tomorrow and 20 Euro in 9 months. All payments were made by checks and given directly (allowance) or sent to participant’s home address either 1 day or 9 months after the date of participation. Checks could be cashed at any bank of choice.

**Table 1:** Descriptive variables of the complete sample and the gender subgroups.

		Age	IQ	Income(year)
**Complete sample**	Mean (SE)	72.6 (0.8)	16.1 (0.6)	22,882 (1,976)
**Males**	Mean (SE)	73.8 (1.1)	16.8 (0.8)	29,409 (2,879)
**Females**	Mean (SE)	71.5 (1.3)	15.4 (0.9)	15,833 (1,890)
**Statistics (m/f)**	t or U	1.369	385^a^	3.942
	*p*	.176	.584	.000**

Means (s.e.m.) of age, IQ score and yearly income. The t or U scores with their *p*-values are reported for the statistical comparison of age, IQ and income between the male and female subgroups.

^a^ Mann-Whitney U test was used instead of t-test due to violation of normality assumption.

** *p* < 0.01.

The study was approved by the ethics committee of the Department of Experimental Psychology at the University of Düsseldorf. Participants were informed about the course of the study, their right to quit the study at any moment for any reason as well as the payment procedure, and all provided written informed consent prior to data collection.

### Materials

#### Intertemporal choice task

Temporal discounting was assessed by a computer-based intertemporal choice task implemented using the MATLAB Toolbox Cogent 2000 (developed by the Cogent 2000 team at the FIL and the ICN). The task consisted of 6 randomized blocks of trials with financial offers that differed in the delays to the smaller, sooner and larger, later reward. In four blocks the delay to the sooner option was *tomorrow*, while the delay to the later option was 3, 6, 9 or 12 months. In the remaining two blocks the sooner option was delayed for 6 months, while the later option was delayed for 9 (block 5) or 12 months (block 6). The delay of the soonest reward was set to *tomorrow* instead of *today* to prevent potential effects of transaction costs on decisions; the expectation of receiving cash payment directly instead by check might bias choices towards immediate rewards. Within each block the later option was fixed at 20 Euro, whereas the sooner option varied between 0 and 20 Euro in steps of 2.50 Euro. Each of the immediate reward values was presented twice within each block, yielding 18 trials per block and 108 trials in total.

Trials in which the immediate reward was either 0 or 20 Euro functioned as *catch* trials, as the preference within these trials should logically be the delayed (e.g., €0 now versus €20 in six months), or immediate reward (e.g., €20 now versus €20 in six months), respectively. Within each trial one option was shown on the left side of the screen (53 cm x 30 cm), while the other was shown on the right side. The side-allocation of smaller-sooner or larger-later rewards was randomized across trials. Choices were indicated by pressing either the ‘x’ or ‘m’ key on a standard keyboard, corresponding to the left or right option shown. The relevant keys were color-coded. Participants received detailed oral and visual instructions before the task was started. It was emphasized that there were no right or wrong answers and their personal preference should guide their decisions. Before the start of each new block the subsequent delays were shown. Participants were told that one of the trials would be randomly picked and their choice paid out by means of checks after the corresponding delay. The final screen of the task displayed their earnings.

#### Episodic memory performance

Episodic memory can be defined as the conscious recollection of past personal events linked to a particular temporal and spatial context (e.g. [54]). In the literature, episodic memory is often decomposed into several sub-functions: associative memory, autobiographical memory for personal events, and autobiographical memory for personal facts and dates. Associative memory is the ability to learn and remember the relationship between unrelated items and is known to rely strongly on the hippocampus [55–57], autobiographical memory refers to the 'meaningful reconstruction of one's own past' [58]. Although autobiographical memory for facts and dates shares the personal component with episodic memory, as well as neural correlates in specific operationalizations (see [59]), it has also been related to semantic memory, and termed 'personal semantics' [59]. We assessed memory performance using independent standard tests for associative and autobiographical memory, as explained in the following paragraphs.

#### Associative memory task

**Fig 1:**
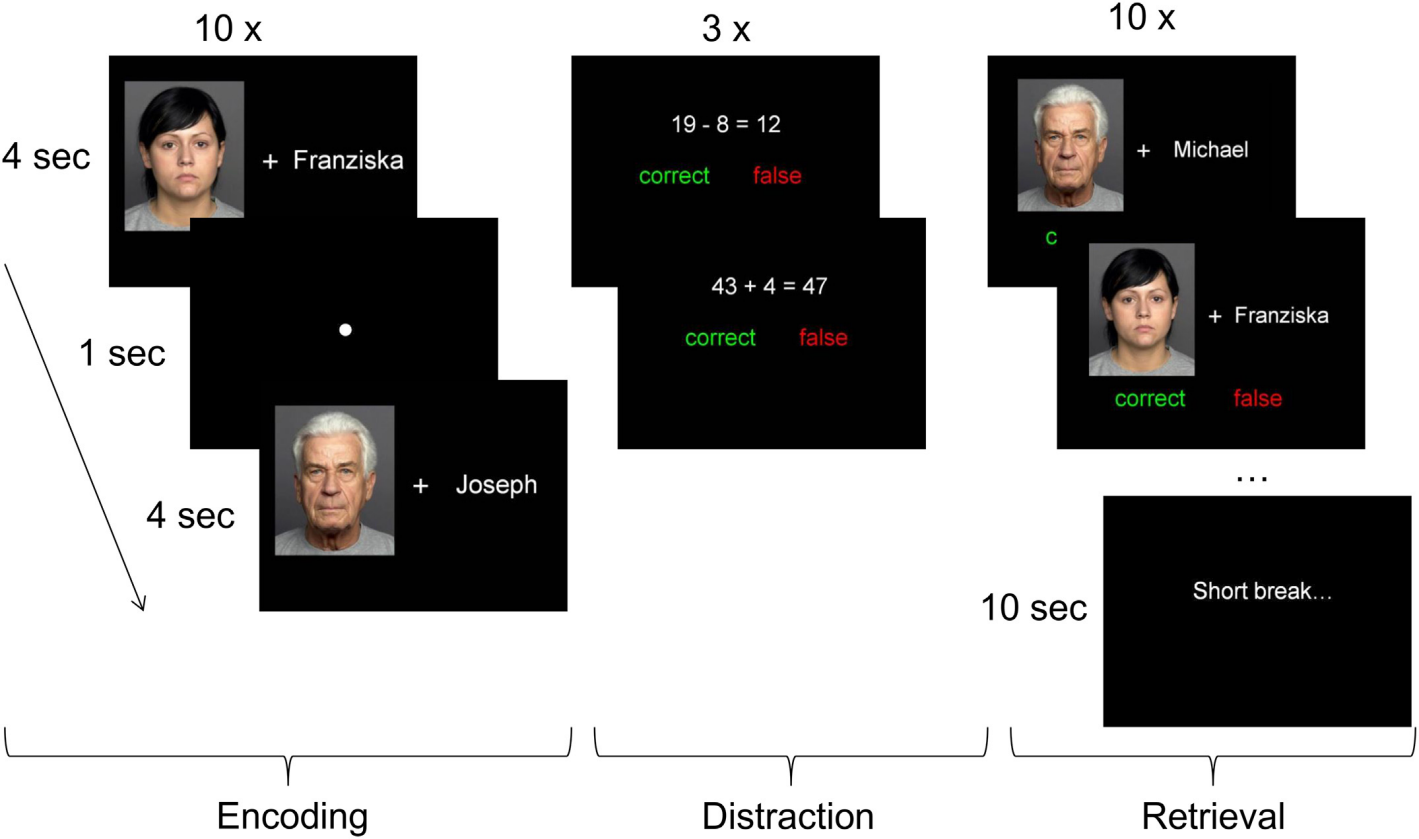
Schematic overview of the Face-Name Paired-Associates (FNPA) task. Participants went through four blocks, consisting of encoding, distraction and retrieval trials. At the beginning of each block participants were required to memorize ten face-name pairs. These were followed by three distraction trials, in which participants had to indicate whether the shown equation was correct or false. Each block ended with ten retrieval trials, in which participants indicated whether the shown face-name pair was correct or false. The faces shown in the figure are from two exemplary persons of the FACES database [60].

Associative memory performance was measured with a face-name paired-associates (FNPA) task, in which new face-name associations need to be stored in memory and subsequently remembered. This task was implemented using the online survey tool Unipark (QuestBack GmbH, Hürth, Germany). It consisted of 4 blocks, each with 10 encoding, 3 subsequent distraction and 10 retrieval trials. The encoding trials consisted of 10 face-name pairs successively presented for 4 seconds each, followed by a fixation dot for 1 second (see Fig 1). Participants were instructed to memorize the name belonging to each face. The face-name pairs were only shown once in random order and were not repeated across blocks. To prevent rehearsal, the encoding trials were followed by the distraction trials, which consisted of mathematical equations presented with a solution that was either correct or false. In each trial participants had to indicate by a mouse click whether the proposed solution was correct or false. In the following retrieval block ten face-name pairs were presented in randomized order, consisting of the same names and faces as shown during the previous encoding trials. However, only half of the trials showed the correct face-name combinations from the first round, the other half contained incorrect face-name combinations. In each trial participants had to indicate with a mouse click whether the face-name pair was *correct* or *false*. Blocks were intermitted by a short break of 10 seconds. Before starting the experiment participants received detailed instructions and performed a test trial to practice handling the mouse and to make sure that they had understood the instructions. In addition, block-specific instructions were shown at the beginning of each block.

The 40 face stimuli used in this task were taken from the FACES database [60] and were unknown to the participants. The stimuli included 14 young (19–28 years), 12 middle-aged (39–54 years) and 14 older (69–80 years) faces with an equal number of male and female faces from each age group and within each block. The fictional names were taken from public lists of popular German forenames and were carefully chosen and assigned to the faces so that they were not suggestive of the person’s age.

#### Autobiographical memory task

The participants’ ability to remember episodes from their past was tested using Module C of the *Inventar zur Gedächtnisdiagnostik* (IGD, [61]) which is a German memory inventory. While Modules A and B measure memory retention and semantic memory, Module C was specifically designed to capture autobiographical memory. Module C again is divided into two sub modules, C1 and C2. While C1 measures memory performance and memory quality for personal events, C2 captures memory performance and quality for dates and facts related to oneself and one’s personal environment.

Sub-module C1 required participants to describe several personal events that occurred before the age of 6, between the age of 7 and 16, from the age of 17 until one year ago and within the last 12 months. The described events must be concrete (i.e. restricted in time and location). For example, having been at college was not counted as an event, whereas the first party at college was. Participants were instructed only to recall events they really *remembered* and not just *knew* from photos, movies or narratives. To ensure that the recalled events fulfilled the requirements and avoid biases due to differences in writing speed, sub module C1 was implemented as interview. A time limit was set to 4 minutes, in which the participants had the chance to describe a maximum of 5 events per life episode. The interviewer was instructed not to prompt or give any hints to facilitate recall, and wrote down the recalled events in note form. Before continuing to the next episode, participants rated one of the recalled events on its *vividness*, *specificity* and *emotionality*, each on a 4-point scale. The to-be-rated event was specified in the test.

Submodule C2 contained 64 items about 11 different general life-related topics (i.e. work, transportation, education). For each item participants had to indicate by ticking ‘yes’ or ‘no’ whether they were able to remember a certain date or fact like, for instance, their partner’s or child’s date of birth, previous home addresses or how they used to travel to school or work. If participants answered ‘yes’ they additionally had to rate how confident they were about the specific memory. Questions about topics that did not apply to the participant (e.g. questions about children with childless subjects) were skipped. There was no time restriction for this part of the IGD.

Although Baller et al. [61] evaluate the content validity of Module C to be sufficient, it should nevertheless be admitted that the participants’ descriptions, answers and ratings could not be verified and were therefore interpreted with caution.

#### General level of intelligence

To control for potential confounding effects of general intelligence, the 10-Minutes-Test [62] was used as a short measure of fluid as well as crystallized intelligence. The test includes 32 items that require mathematical and deductive reasoning or general knowledge and vocabulary. The total test score corresponds to the sum of all items that were solved correctly within 10 minutes. This relatively short implementation time allows for a fast, yet valid and reliable estimate of general intelligence. Beside its objective administration and interpretation, the test was shown to have a high loading on Spearman’s g factor with r = .57 [63]. First validation studies also found a high internal consistency of Cronbach’s α > .80 and significant correlations with several other cognitive measures [62, 64]. To date the 10-Minutes-Test has only been standardized for pupils and students. However, due to the lack of alternative short intelligence screening methods and the possibility that a long test session might overstrain the cognitive capacity of older participants, the 10-Minutes-Test was considered to be suitable for our purposes.

#### Dementia

Participants were screened for dementia or severe cognitive impairment using the mini-mental state examination (MMSE). This interview assesses global cognitive abilities like orientation, attention and memory as well as numerical and language skills. Participants with scores lower than 13 out of 30, which indicate global cognitive disorders, were excluded from participation. All screened participants had a score above 13.

#### Post-test questionnaire

General demographic information (education, job status, income and lifestyle habits) was obtained with a post-test questionnaire.

### Procedure

The experiment took place in a laboratory at Heinrich-Heine-University Düsseldorf. Participants were tested individually in one 90 minute session. Participants were given verbal and written information on the procedure of the experiment before giving informed written consent. All participants were screened for dementia before performing the tasks. The order of tasks was determined depending on their importance and degree of difficulty, with the most demanding tasks set at the beginning. Thus, participants started with the intertemporal choice task, followed by the FNPA task, the 10-Minutes-Test, and finally the IGD task. In the end, participants filled out the post-test questionnaire and received their general show-up fee of 5 Euro. In addition, they were given a signed receipt stating that the remaining amount earned in the intertemporal choice task, to be received at the specified date, would be sent in the form of a check by post.

### Data analysis

#### Delay discounting

All mathematical procedures to determine the participants’ discount rates were performed using the MATLAB (The MathWorks, Inc.). First of all, we identified, for each of the six blocks, the individual indifference points (IPs; the amount for the smaller, sooner reward that renders the smaller, sooner reward equally valuable as the larger, later reward) using logistic regression. For further analysis, all IPs were converted into proportions of the late reward of 20 Euro.

We fitted two different models to the estimated IPs of blocks 1 to 4. First, we fitted the standard hyperbolic model [1, 65, 66]:

$$SV_{\text{T}}\;=\;A\;/\;\left(1\;+\;kT\right)$$ (1)

where *SV* is the subjective value of the reward, *A* is the monetary amount of the reward and *T* is the delay in months. The amount was set to *A* = 1 as the values were expressed as proportions of the later reward. Larger k-values indicate a greater impact of delay on value and therefore steeper discounting.

In addition, Laibson’s [67] quasi-hyperbolic β-δ model was fitted to the indifference point to obtain measures of present-bias and patience:

$$SV_{\text{T}}{}_{=0}=\;1$$

$$SV_{\text{T}>0}\;=\;\beta *\;\delta^{\text{T}}$$ (2)

*SV*_*t*_ is the subjective value of a reward at time *T*. This equation models the often found initial rapid decline in subjective value with small delays (present-bias) separately, represented by the parameter β (with 0 ≤ β ≤ 1). The inverse of β can be interpreted as the extra weight added to immediacy, thus smaller β-values can be construed as stronger present-bias. In our analysis, T = 0 corresponds to ‘tomorrow’, as this was the soonest option available in our task. We opted to define tomorrow, and not today, as the soonest option to control for potential transaction costs, and assumed that tomorrow would be part of the extended present [68]. The discount function’s discount rate is log(1/δ). Thus, the parameter δ (with 0 ≤ δ ≤ 1) can be interpreted as a measure of patience with higher δ-values indicating higher patience.

**Fig 2:**
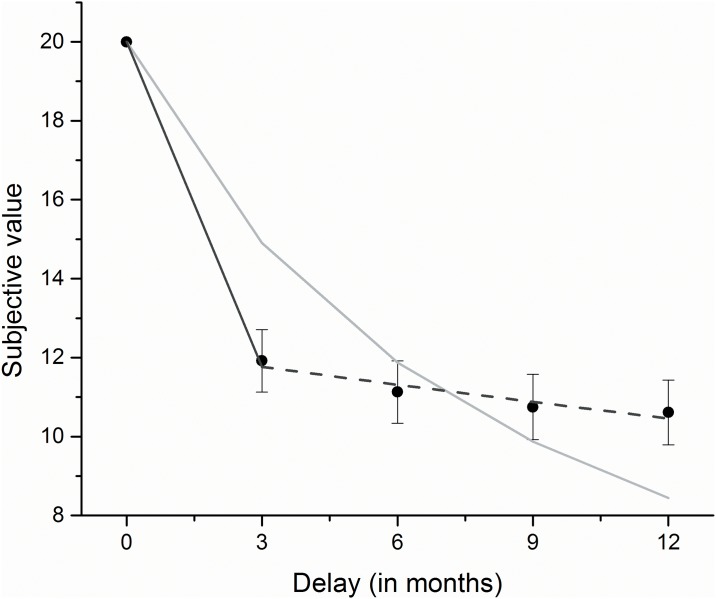
Illustrative hyperbolic and quasi-hyperbolic model fit to the average choice data of the whole sample. The indifference points at 3, 6, 9 and 12 months and one day, averaged across participants, were used to fit the hyperbolic model (light gray line) and the quasi-hyperbolic model (dark gray and dashed line). Errorbars show the standard error of the mean (s.e.m.). The steepness of the hyperbolic function is reflected by parameter *k*. The dark grey line of the quasi-hyperbolic model represents present-bias and is reflected by parameter *β*, whereas further decline in value (dashed line) is reflected by ‘patience’ parameter *δ*.

The hyperbolic and quasi-hyperbolic models were fit to the first four indifference points, which were implemented as proportions of the delayed reward (e.g. an indifference point of 10 Euro would yield a proportion of .5 relative to an immediately available 20 Euro reward) for each participant individually, using a least-squares algorithm implemented in MATLAB R2013a (The MathWorks, Inc.). The fitting parameters *k*, *β* and *δ* were allowed to vary freely. Fig 2 shows the average indifference points of the whole sample as well as their hyperbolic and quasi-hyperbolic fits. Goodness of fit analyses using the Akaike Information Criterion (AIC), which takes into account the number of parameters, showed that the data was better described by Laibson’s quasi-hyperbolic model (M = -27.8) than the standard hyperbolic model (M = -11.5).

We furthermore conducted additional analyses with several model-free parameters, which yielded similar results (see S1 Text).

#### Associative and autobiographical memory performance

The four retrieval blocks of the FNPA task contained 20 correctly and 20 falsely paired face-name pairs. Correct face-name pairs that were recognized as such were counted as hits (FNPA-Hits), whereas face-name pairs that were actually false but judged as correct were counted as false alarms (FNPA-FA). Overall associative memory performance (FNPA-PF) was calculated by subtracting false alarms from hits (FNPA-PF = FNPA-Hits—FNPA-FA).

Module C of the IGD was analyzed according to the standard procedure suggested in the test manual [61]. The overall score of sub module C1 (IGD-C1) consists of the total number of events recalled proportional to the maximum of 20 recalled events, added to the sum of the quality rating scores from all four episodes proportional to the maximum rating score of 36. The score of sub module C2 (IGD-C2) is the proportion of all items answered with *yes* relative to the total number of answered items added to the proportion of quality rating scores from all answered areas.

#### Statistical analyses

Statistical analyses reported below were performed using the software package IBM SPSS Statistics 20. The main analysis consisted of OLS regressions using the discounting parameters as dependent variables and the memory scores and mediator/moderator variables age, IQ and income as predictors. We used three models. In the first model,

$$Discounting\;parameter\;=\;b_{\text{0}}\;+\;b_{\text{1}}*\text{FNPA-PF}\;+\;b_{\text{2}}*\text{IGD-C1}\;+\;b_{\text{3}}*\text{IGD-C2}$$ (3)

we check for the effects of the memory scores on the discounting parameters. In the second model the mediator/moderator variables and gender were added:

$$Discounting\;parameter\;=\;b_{\text{0}}\;+\;b_{\text{1}}*\text{FNPA-PF}\;+\;b_{\text{2}}*\text{IGD-C1}\;+\;b_{\text{3}}*\text{IGD-C2}\;+\;b_{\text{4}}*Gender\;+\;b_{\text{5}}*age\;+\;b_{\text{6}}*IQ\;+\;b_{\text{7}}*income$$ (4)

In the third model, three interaction terms of gender and memory performance were added:

$$Discounting\;parameter\;=\;b_{\text{0}}\;+\;b_{\text{1}}*\text{FNPA-PF}\;+\;b_{\text{2}}*\text{IGD-C1}\;+\;b_{\text{3}}*\text{IGD-C2}\;+\;b_{\text{4}}*Gender\;+\;b_{\text{5}}*age\;+\;b_{\text{6}}*IQ\;+\;b_{\text{7}}*income\;+\;b_{\text{8}}*\left(\text{FNPA-PF}*Gender\right)\;+\;b_{\text{9}}*\left(\text{IGD-C1}*Gender\right)\;+\;b_{\text{10}}*\left(\text{IGD-C2}*Gender\right)$$ (5)

Missing data (see results) was replaced using the Expectation-Maximization procedure [69] to ensure inclusion of all participants in the regression analyses.

In addition, several correlation analyses were performed (see supplemental material). Where necessary, the significance level α was adjusted using the Holm-Bonferroni method to control the familywise error rate.

## Results

We excluded participants from further analysis when more than half of the *catch trials* in the intertemporal choice task were answered incorrectly. We assumed that this indicated insufficient attention or understanding of the task. Four participants met this criterion and were therefore excluded from further analyses, rendering the overall sample size at *n = 58*. However, the main results reported in the following sections did not change when these participants were included. Furthermore, *income* data was missing in 6 participants.

Since we also found gender effects (see below), results are presented for the complete sample as well as the male (*n = 28*) and female (*n = 30*) subsamples. Table 1 summarizes the descriptive variables *age*, *IQ* and *income* for the complete, male and female group. The gender subgroups only differed in their income, with the women subgroup earning significantly less than men, *t(56) = 3*.*942*, *p <* .*001*. In addition, Table 2 shows the averages and standard deviations for the memory task scores and the discount parameter values, for the complete group as well as the gender subgroups. As predicted, women scored higher than men on the episodic memory tasks, but there was no significant gender difference in discount behavior. Correlations of the different episodic memory scores for men and women are summarized in S2 Table.

**Table 2:** Intertemporal choice and episodic memory scores of the complete sample and the gender subgroups.

		FNPA-PF	IGD-C1	IGD-C2	k	β	δ
**Complete sample**	Mean (SE)	0.45 (0.03)	1.68 (0.03)	1.72 (0.03)	0.36 (0.08)	0.65 (0.04)	0.98 (0.00)
**Males**	Mean (SE)	0.33 (0.04)	1.62 (0.05)	1.66 (0.04)	0.29 (0.09)	0.67 (0.05)	0.98 (0.01)
**Females**	Mean (SE)	0.57 (0.04)	1.74 (0.03)	1.77 (0.03)	0.43 (0.12)	0.62 (0.05)	0.97 (0.01)
**Statistics (m/f)**	t or U	-4.322	-2.126	-2.130	-.784^b^	.716	416 ^a^
	p	.000**	.038*	.038*	.437	.477	.950

Means (s.e.m.) of the episodic memory scores and the discount parameters for the complete sample as well as the gender subgroups. The t or U scores, as well as the *p*-values are reported for the comparison of the task scores between the male and female subgroups.

^a^ Mann-Whitney U test was used instead of t-test due to violation of normality assumption.

^b^ Ln(k) was used to test difference between male and female subgroups.

* *p* < 0.05.

** *p* < 0.01.

Since we expected our participant sample to show variable memory scores that would reflect the level of cognitive decline related to healthy aging, we checked for correlations between the memory scores *FNPA-PF*, *IGD-C1* and *IGD-C2* and *age*, as well as *IQ* and *income*. Results are shown in Table 3. Only the *FNPA-PF* scores showed a close-to-significant negative correlation with *age*, *r* = -.*308*, *p =* .*018 > α =* .*017*, *r* ^*2*^ *=* .*09*, suggesting that older participants have lower scores on the FNPA task.

**Table 3:** Correlations of memory scores with age, IQ and income within the complete sample.

	Age	IQ	Income
**Associative memory**			
** FNPA-PF**	-.308 (.018)	.248 (.061)	-.243 (.082) ^a^
**Autobiographical memory**			
** IGD-C1**	-.062 (.644)	-.061 (.649)	-.019 (.893) ^a^
** IGD-C2**	-.201 (.130) ^a^	.190 (.154) ^a^	-.141 (.318) ^a^

Correlation coefficients and p-values (in brackets) of the correlations of episodic memory scores and mediator/moderator variables, using the complete sample. All p-values are two-tailed.

^a^ Spearman’s Rho was used due to violation of normality assumption.

### Regression analyses

Separate regression analyses with the discounting parameters *ln(k)*, *β* and *δ* as dependent variables and memory scores (*FNPA-PF*, *IGD-C1* and *IGD-C2*) as predictors show no significant contribution of any memory score on the model-based discounting values on group level, *ln(k)*: *F(3*,*54) =* .*153*, *p =* .*928*, *R* ^*2*^ *=* .*008; βF(3*,*54) =* .*067*, *p =* .*977*, *R* ^*2*^ *=* .*004; δ*:*F(3*,*54) =* .*272*, *p =* .*846*, *R* ^*2*^ *=* .*015* (Table 4). Thus, our results did not support the hypothesis that a better functioning episodic memory system is associated with reduced discounting.

**Table 4:** Relationship of memory scores, moderator variables and gender x memory interactions with discounting parameters.

	Ln(k)			β			δ		
*Model*	*(1)*	*(2)*	*(3)*	*(1)*	*(2)*	*(3)*	*(1)*	*(2)*	*(3)*
**FNPA-PF**	-.087 (.545)	-.140 (.348)	.098 (.828)	.023 (.872)	.025 (.883)	-.253 (.575)	.106 (.459)	.211 (.241)	.349 (.499)
**IGD-C1**	.045 (.755)	.042 (.758)	-.218 (.601)	-.047 (.741)	-.039 (.786)	-.074 (.860)	-.033 (.818)	-.028 (.855)	.934 (.055)
**IGD-C2**	.033 (.822)	.032 (.819)	-1.032 (.015)*	.045 (.762)	.029 (.842)	1.389 (.002)**	-.087 (.555)	-.056 (.719)	-.249 (.600)
**Gender**		-.026 (.882)	-.054 (.741)		.038 (.833)	.088 (.596)		-.126 (.508)	-.170 (.370)
**Age**		.119 (.387)	.122 (.354)		-.151 (.289)	-.146 (.271)		.018 (.902)	-.015 (.922)
**Income**		-.427 (.005)*	-.382 (.009)**		.326 (.036)*	.298 (.041)*		.082 (.611)	.008 (.962)
**IQ**		.000 (.999)	-.109 (.913)		.059 (.691)	.105 (.455)		-.106 (.498)	-.182 (.255)
Gender*FNPA-PF			-.248 (.576)			.267 (.550)			-.082 (.872)
Gender*IGD-C1			.358 (.383)			-.069 (.866)			-.977 (.038)*
Gender*IGD-C2			1.129 (.007)**			-3.538 (.001)**			.138 (.764)
***F Statistic (df)***	.*153 (3*,*54)*	*1*.*741 (7*,*50)*	*2*.*374 (10*,*47)*	.*067 (3*,*54)*	*1*.*158 (7*,*50)*	*2*.*285 (10*,*47)*	.*272 (3*,*54)*	.*292 (7*,*50)*	.*673 (*.*10*,*47)*
***R*** ^***2***^	.*008*	.*196*	.*579*	.*004*	.*139*	.*327*	.*015*	.*039*	.*125*
***Adjusted R*** ^***2***^	*-*.*047*	.*083*	.*194*	*-*.*052*	.*019*	.*184*	*-*.*040*	*-*.*095*	*-*.*061*
***p-value***	.*928*	.*121*	.*023* *	.*977*	.*344*	.*028* *	.*846*	.*954*	.*743*

Each column represents one OLS regression. Dependent variables are shown in the column titles. Each row represents one predictor variable or statistics of the regression model. Cells show regression coefficients (beta) and p-values in brackets or model statistics.

* p < .05.

** p < .01.

The second regression model showed no significant contribution of *gender*, *age* or *IQ* on discounting parameters *ln(k)*, *β* and *δ*, whereas the variable *income* significantly predicted *ln(k)* (*beta = -*.*427*, *p =* .*005*) and *β* (*beta =* .*326*, *p =* .*009*) (Table 4). A higher income was related to lower values of *k* and thus less discounting. This was reflected by a similar significant effect of *income* on the *number of impulsive choices (NImp)* (see S1 Table). Similarly, a higher income was associated with higher values of *β*, indicating a lower present-bias.

**Fig 3:**
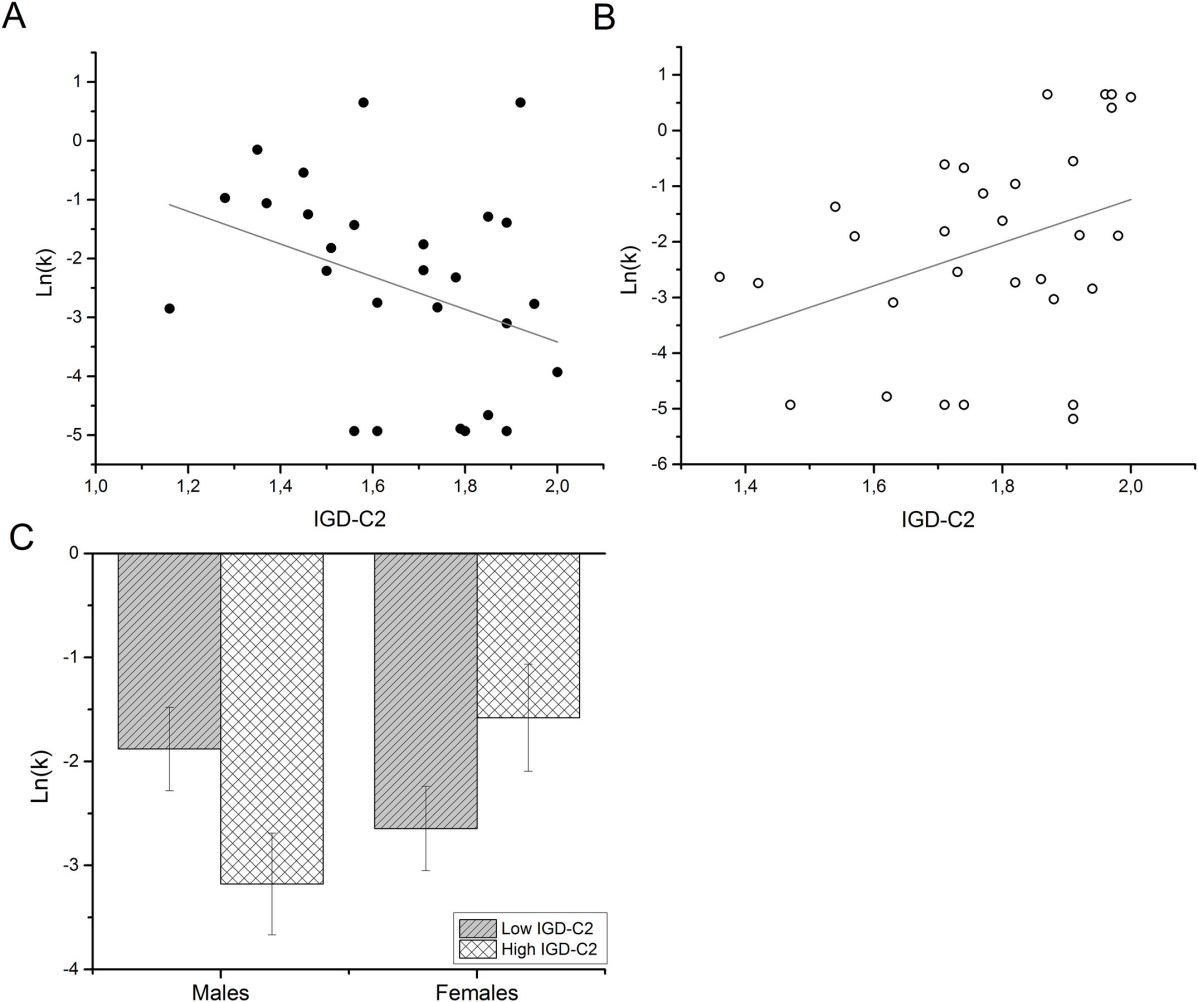
Interaction effects of gender and *IGD-C2* scores on ln(*k*). (A) Scatterplot with regression line of the IGD-C2 and ln(*k*) scores in the male subsample. (B) Scatterplot with regression line of the IGD-C2 and ln(*k*) scores in the female subsample. (C) To further illustrate the gender-dependent differences in the relationship between memory performance and discounting, we performed a median split to categorize participants according to their IGD-C2 performance (high- vs. low performers). Individual bars show mean ln(*k*) values for subgroups with high and low IGD-C2 scores. Error bars show the standard error of the mean (s.e.m.).

**Fig 4:**
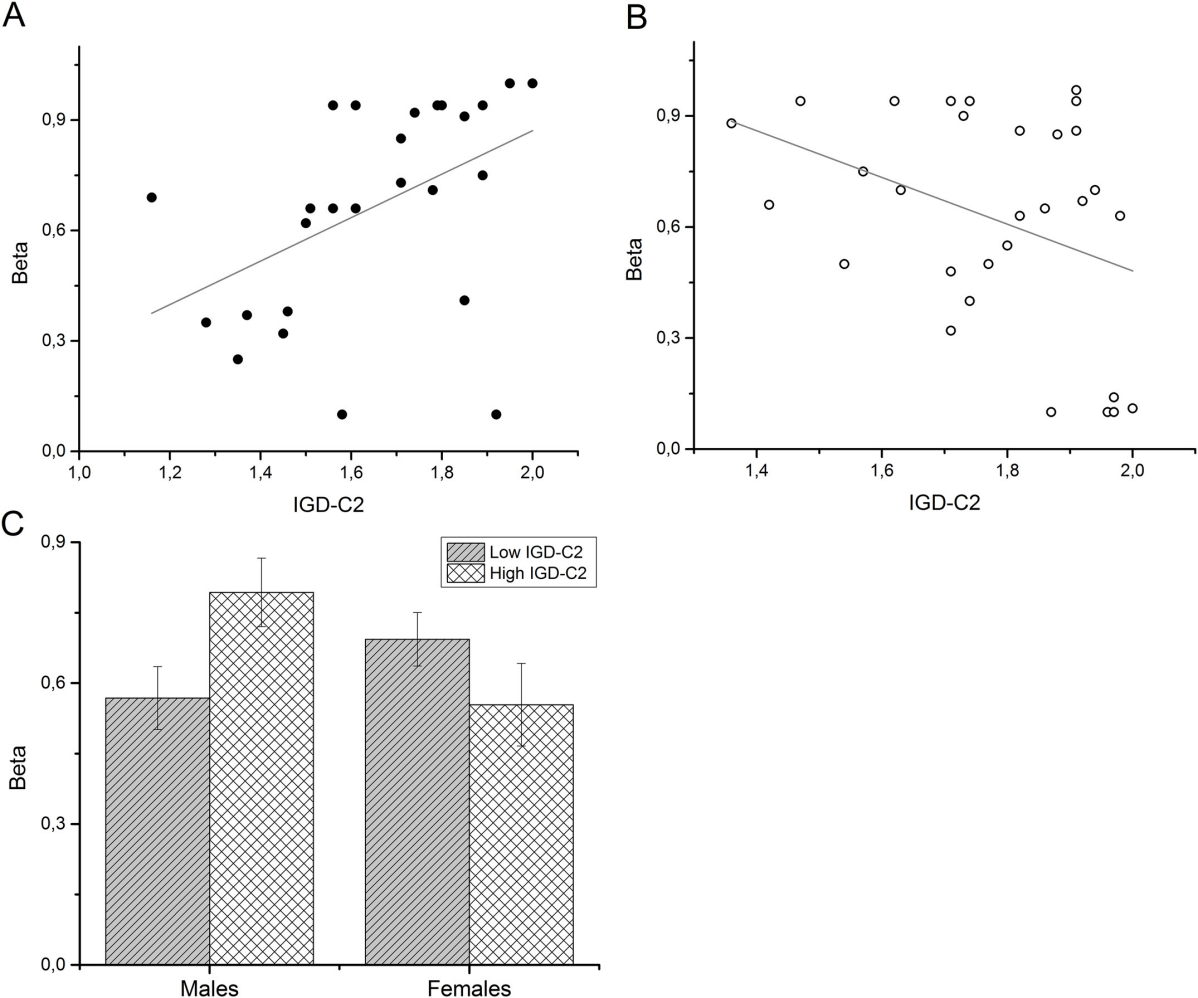
Interaction effects of gender and IGD-C2 scores on parameter *β*. (A) Scatterplot with regression line of the IGD-C2 and *β*scores in the male subsample. (B) Scatterplot with regression line of the IGD-C2 and *β*scores in the female subsample. (C) To further illustrate the gender-dependent differences in the relationship between memory performance and discounting, we performed a median split to categorize participants according to their IGD-C2 performance (high- vs. low performers). Individual bars show mean *β*values for subgroups with high and low IGD-C2 scores. Error bars show the standard error of the mean (s.e.m.).

To further explore a potential effect of gender, interaction terms were calculated by multiplying centered memory scores with gender. The addition of the three interaction terms (*gender* x *FNPA-PF*, *gender* x *IGD-C1* and *gender* x *IGD-C2*) in the third regression model revealed several interaction effects (Table 4). The interaction between *gender* and *IGD-C2* scores significantly predicted discount parameters *ln(k)* (*beta = 1*.*129*, *p =* .*007*) as well as *β* (*beta = -3*.*538*, *p =* .*001*) (Table 4), indicating that in males, a higher scores for autobiographical fact and date recall went along with less discounting, whereas in females higher recall scores went along with more discounting (see Fig 3 and Fig 4). The increase in *R* ^*2*^ was significant compared to the second regression model for both *ln(k)*, *F(3*,*47) = 3*.*292*, *p =* .*029*, and *β*, *F(3*,*47) = 4*.*371*, *p =* .*009*, as dependent variable. As gender is a binary variable, there was a high correlation between the interaction terms and the corresponding memory terms. Therefore, the significant effects of the memory measures in regression model 3 should be ignored.

**Fig 5:**
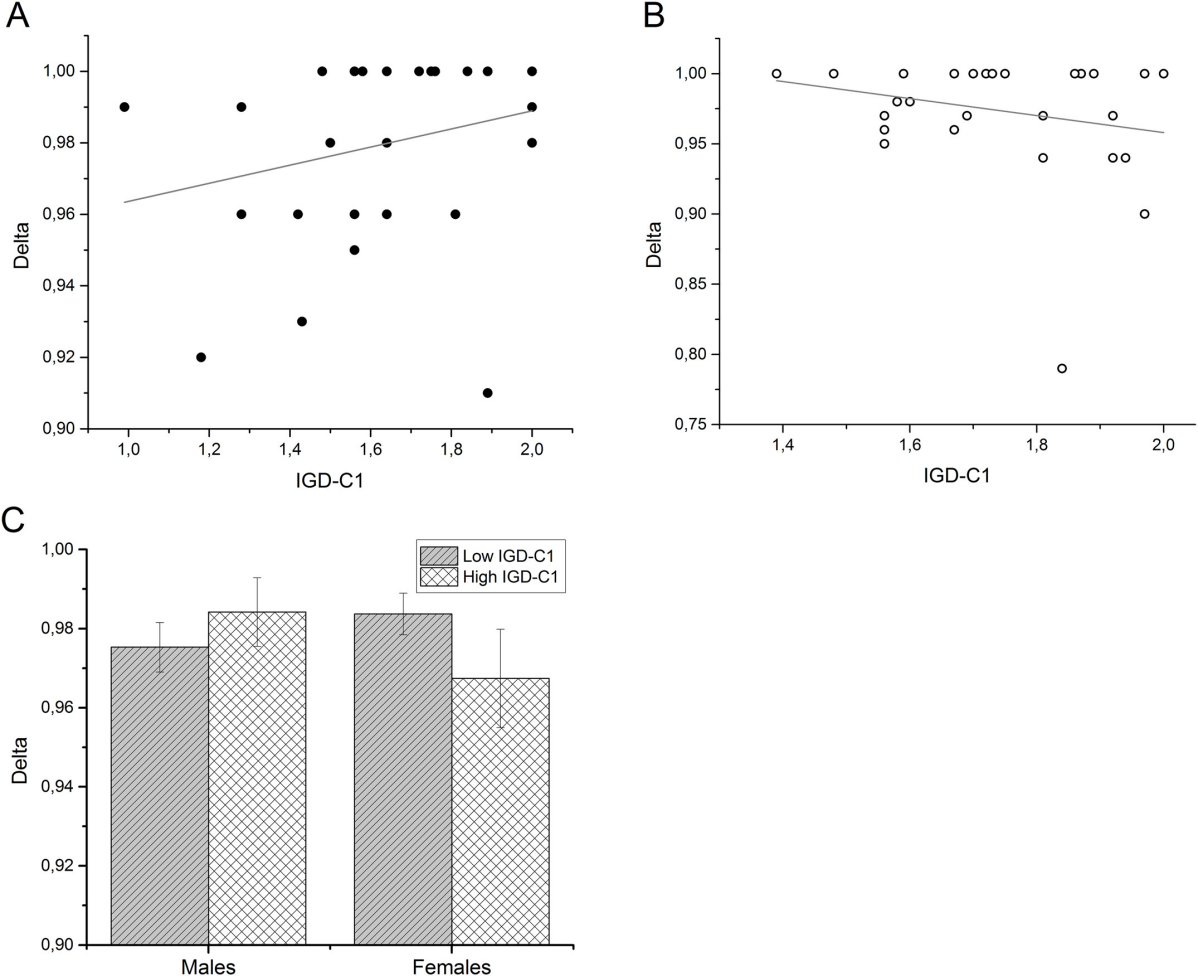
Interaction effects of gender and IGD-C1 scores on parameter *δ*. (A) Scatterplot with regression line of the IGD-C1 and *δ* scores in the male subsample. (B) Scatterplot with regression line of the IGD-C1 and *δ* scores in the female subsample. (C) To further illustrate the gender-dependent differences in the relationship between memory performance and discounting, we performed a median split to categorize participants according to their IGD-C1 performance (high- vs. low performers). Individual bars show mean *δ* values for subgroups with high and low IGD-C1 scores. Error bars show the standard error of the mean (s.e.m.).

Further, a similar significant interaction was found for *IGD-C1* scores and the discounting parameter *δ* (*beta = -*.*977*, *p =* .*038*) (Table 4), indicating that better recall of autobiographical events went along with more patience in males, whereas in females better autobiographical memory predicted more impatience (see Fig 5). However, the model itself as well as the increase in *R* ^*2*^ was not significant, *F(3*,*47) = 1*.*540*, *p =* .*217*.

## Discussion

The aim of this experiment was to investigate the relationship between episodic memory and delay discounting in older adults. We hypothesized that episodic memory performance correlated with intertemporal choice behavior. Overall, we found no evidence for a relationship between episodic memory scores and time discounting. However, when considering gender differences, we found several interactions of gender and memory scores on discounting. First of all, we found that higher memory scores for autobiographical facts and dates, but not associative memory performance or memory for personal events, were related to a decreased level of discounting in men, as indicated by lower values of the hyperbolic discounting parameter *k* and higher values of present-bias parameter *β*. By contrast, in women, we found the opposite pattern; higher autobiographical memory scores for facts and dates were related to higher levels of discounting, as indicated by higher values of *k* and lower values of *β*. Furthermore, a similar interaction was found for gender and recall of autobiographical events on the discounting parameter *δ*, representing patience. Whereas men with better autobiographical event recall showed more patience, women with better autobiographical event recall showed less patience. These findings were not due to a general difference in discounting behavior between men and women.

Our hypothesis that episodic memory functioning and time discounting may be related was based on the finding that the core network involved in episodic memory functioning is also responsible for episodic future thinking, which has been related to lower rates of discounting [33–35], e.g. through episodic tagging techniques. Here, we focused on memory retrieval of past episodes and events instead of eliciting future thinking. The question remains whether participants still use future simulation during intertemporal choice when not explicitly elicited.

If future rewards are indeed imagined when not being explicitly triggered during temporal discounting, one explanation of our findings could be that imagining future outcomes might depend on recall of past personal facts, rather than events, to form a new ‘image’ of the future. Although we do not find a general effect of autobiographical memory on discounting, we do find interaction effects of gender with autobiographical memory recall on discounting, with recall of events linked to patience, and recall of facts and dates linked to present-bias as well as the overall level of discounting.

Present-bias can be characterized using Laibson’s quasi-hyperbolic model [67] as the drop in subjective value between obtaining the monetary reward now and obtaining it in the nearest possible future. In contrast, the level of patience (reflected by parameter *δ*) gives an indication of how further delays affect the subjective value of the reward. Present-bias can be seen as a measure of intertemporal inconsistency, as a stronger present-bias indicates a larger deviation from constant time discounting (e.g. a linear or exponential decrease in value of a reward with time), due to a disproportionally strong focus on the present. That personal semantics might have a particular effect on present-bias makes sense with regard to the semantic content of any imagined future event, which would be similar regardless of the specific delay associated with it. As the parameter *δ* indicates further decline of value with increasing delays, this parameter is sensitive to the temporal context, which is an important aspect of a particular event and thus of episodic memory. It therefore makes sense that this parameter is related to recall of autobiographical events.

Previous literature has shown not only a dissociation between memory for autobiographical events and autobiographical facts and dates on behavioral level [70, 71], but also in underlying brain mechanisms [72, 73]. Just as retrieval of general facts and events, the retrieval of autobiographical facts is less dependent on the connectivity between the parahippocampal gyrus and hippocampus than the recall of autobiographical events [73]. Hence, recall mechanisms for autobiographical facts might have more in common with recall of general facts and events, which is less hippocampus-dependent, and less with recall of specific autobiographical events. For example, the recall of the means of transportation during one’s first job does not require recall of a specific time and space. Indeed, the memory of autobiographical facts and dates is also termed 'personal semantics' [59]. The specificity of the relationship between recall of autobiographical facts and dates and present-bias might therefore depend more on general memory retrieval mechanisms, and less on mechanisms specific for retrieval of highly context-dependent memories, such as autobiographical events.

That other (i.e. semantic or unconscious) long-term memory mechanisms could play a role is also supported by the absence of a relationship between associative memory performance and discounting. It is thought that with healthy aging, primarily the formation of new memories is affected, whereas older episodic memories are relatively preserved [74, 75]. The autobiographical memory task used here probably depends less on hippocampal functioning and more on neocortical integrity, as memory retrieval of items stored long ago in general seems to depend more on the latter [76, 77], whereas the associative memory task used here assessed both the formation and recall of newly formed face-name associations, which might rely most on hippocampal areas affected early with aging. In line with this view, we found a close-to-significant negative correlation between associative memory (FNPA) scores and age, but not between autobiographical memory scores and age. Why would older-aged men and women with better recall of autobiographical facts and dates/events show such opposing patterns regarding their present-bias/patience? It is possible that men differ from women regarding their “cognitive style” by which they make economic decisions. In a fMRI study by Piefke et al. [78], three brain areas were found to exhibit differential responses in men and women during autobiographical memory retrieval; whereas the parahippocampal region was more active in men, the right dorsolateral prefrontal cortex (dlPFC) as well as the right insular cortex showed increased activity in women compared to men. More recently, Young et al. [79] replicated the finding that women showed increased activity in the right dlPFC during autobiographical memory recall. Since there were no gender differences in behavioral performance, these findings likely support the “cognitive style hypothesis”, which states that men and women differ in the way they encode, rehearse and process emotional experiences, and exhibit differential response strategies during laboratory memory tasks [77, 79, 80].

The prefrontal cortex (PFC) is one of the areas found to be important for episodic memory retrieval, and PFC functioning is related to recalling the temporal context of memories [81–85]. An fMRI study by Suzuki et al. [85] indicated that the right dlPFC is predominantly engaged in recall of the temporal order of separate events, whereas the left dlPFC showed more engagement in recall of the temporal order within a specific event. It was therefore argued that the increased activity in the right dlPFC in women compared to men might reflect that women relied more strongly on the temporal context of autobiographical memories when recalling these events [77]. If this is indeed the case, it is possible that women are differentially sensitive to temporal information when it comes to imagining future rewards. This might explain why women with better recall for *past* autobiographical facts/events showed more sensitivity for the delays to *future* outcomes in our task, and as a result showed increased discounting.

The parahippocampal region has been shown to be involved in spatial learning, navigation and spatial context memory [86–88]. In men, the increased activity found in this area during episodic memory tasks compared to women [78] could therefore point towards an increased role of spatial context, not only during recall of autobiographical events, but also when imagining future rewards. A general larger emphasis on spatial context processing in men, in combination with increased dependence of autobiographical memory recall performance on spatial context processing, could potentially explain why men, but not women, with better memory scores showed less discounting. It thus seems that men and women differ in their cognitive styles and strategies when performing episodic memory tasks [77, 79, 89], and arguably this difference might generalize to performance on other tasks requiring putatively similar cognitive strategies, such as future episodic simulation during delay discounting.

It is possible that this gender difference only occurs in older-aged individuals. One of the aims of this study was to investigate whether individual differences in episodic memory functioning and age-related decline could potentially explain the large variability in the effects of aging on time discounting. Although gender effects are not consistently found in time discounting, memory decline could give rise to differential choice preferences in males and females, which can bias the overall picture towards more or less discounting in older adults compared to younger groups. However, more research is necessary to shed light on this issue.

Recent studies [46, 90], in which patients with medial temporal lobe (MTL) lesions that showed impaired episodic future thinking completed an intertemporal choice task, indicated that processes involving the MTL are not essential for discounting, as these amnesic patients had similar discounting rates as control participants [45, 46, 90]. However, when amnesic patients were cued to imagine future events during the intertemporal choice task, they did not show decreased levels of discounting, whereas a healthy control group did show the decreased levels of discounting shown in previous studies [46]. Therefore, processes involving the MTL, such as episodic future thinking, may only play a role as moderators on intertemporal decision making [46, 90] and might therefore not necessarily be invoked by default.

Interestingly, when amnesic patients were asked to imagine personal future situations [90], instead of more general future events [46], they also show the attenuating effect of episodic future thinking on discounting, suggesting that different ‘types’ of future thinking depend more or less on MTL functioning. A study with patients with semantic dementia shows that episodic future thinking is critically dependent on semantic memory, as these patients showed relatively intact episodic memory for recent past events, but impaired episodic future thinking [91]. Kwan et al. [90] suggest this role of semantic memory could have been the cause of the differential effects of their study compared to the results found by Palombo et al. [46]; the personal cues might have triggered semantic future thinking instead of episodic future thinking [90], yielding similar reductions in discounting. This would be in line with our finding that memory for autobiographical facts and dates (i.e. personal semantics) is related to discounting.

Several additional measures were found to differ between the gender groups. In line with previous findings, we found that women scored higher on episodic memory tasks than men [30, 49, 50, 52]. Whether this is due to differences in verbal production/visuospatial processing [52] or possibly a difference in the richness of detail encoding between men and women [80] remains unclear, although our results showed that this gender effect is not limited to autobiographical events, as also associative memory performance showed a gender effect. In contrast, our results revealed no gender- difference in discounting, present-bias or patience in older subjects. Second, men and women differed in their income. This is potentially important as income is found to be related to time discounting [9, 14, 92, 93]. However, our results did not change when including income as additional variable in our partial correlation analyses, suggesting that income affected discounting independent of episodic memory.

In summary, we found no clear evidence for a general relationship between episodic memory and delay discounting in older-aged adults. However, we found a gender difference in this relationship: whereas men with better memory for autobiographical facts and dates/events showed less present-bias/more patience, women with better autobiographical memory were more present-biased/impatient. The finding that older-aged men and women with better autobiographical recall discount less, or more respectively, could be explained by assuming gender-differences in “cognitive styles” when making intertemporal decisions. As these interaction effects were not predicted, further behavioral studies should confirm these findings. Whether this interaction of gender and temporal discounting in older adults depends on neocortical integrity, in particular of the right dlPFC, hippocampus, or entirely different networks, requires verification using additional methods, such as fMRI.

## Supporting Information

S1 Table Relationship of memory scores, moderator variables and gender x memory interactions on model-free discounting measures. Each column represents one OLS regression. Dependent variables are shown in the column titles. Each row represents one predictor variable or statistics of the regression model. Cells show regression coefficients (beta) and p-values in brackets or model statistics. * p < .05. ** p < .01.
(DOCX)

S2 Table Correlations of the different episodic memory tasks. Correlation coefficients and p-values (in brackets) are reported. All p-values are two-tailed. * p < .025.
(DOCX)

S1 Text Supplementary Information. (DOCX)
