# Supplementary material for: Gender-Specific Differences in the Relationship between Autobiographical Memory and Intertemporal Choice in Older Adults
Source: PLoS One. 2015 Sep 3;10(9):e0137061. doi: 10.1371/journal.pone.0137061 (PMC4559386; doi:10.1371/journal.pone.0137061)
Supplement: S1 Table — Each column represents one OLS regression. Dependent variables are shown in the column titles. Each row represents one predictor variable or statistics of the regression model. Cells show regression coefficients (beta) and p-values in brackets or model statistics. * p < .05. ** p < .01. (DOCX) [file pone.0137061.s001.docx]

|  | **NImp** |  |  | **PB-3** |  |  | **PB-6** |  |  |
| --- | --- | --- | --- | --- | --- | --- | --- | --- | --- |
| ***Model*** | *(1)* | *(2)* | *(3)* | *(1)* | *(2)* | *(3)* | *(1)* | *(2)* | *(3)* |
| **FNPA-PF** | -.065 (.652) | -.090 (.578) | .183 (.678) | .080 (.575) | .040 (.822) | -.304 (.538) | -.142 (.308) | -.278 (.107) | .068 (.891) |
| **IGD-C1** | .390 (.698) | .043 (.758) | -.300 (.467) | -.028 (.844) | -.013 (.931) | -.336 (.464) | .180 (.200) | .174 (.229) | -.365 (.431) |
| **IGD-C2** | .030 (.840) | .035 (.804) | -1.007 (.016) | .026 (.862) | .011 (.944) | -.888 (.055) | .136 (.343) | .095 (.518) | -.209 (.647) |
| **Gender** |  | .008 (.963) | .015 (.926) |  | -.097 (.610) | -.111 (.544) |  | .093 (.611) | .101 (.581) |
| **Age** |  | .191 (.163) | .195 (.136) |  | -.131 (.379) | -.094 (.517) |  | -.161 (.263) | -.162 (.270) |
| **Income** |  | -.390 (.010)* | -.338 (.019)* |  | -.232 (.151) | -.176 (.263) |  | -.181 (.244) | -.132 (.404) |
| **IQ** |  | -.034 (.808) | -.043 (.753) |  | -.013 (.931) | -.016 (.914) |  | .044 (.768) | .072 (.640) |
| **Gender*FNPA-PF** |  |  | -.292 (.503) |  |  | .359 (.462) |  |  | -.395 (.423) |
| **Gender*IGD-C1** |  |  | .443 (.275) |  |  | .400 (.377) |  |  | .597 (.193) |
| **Gender*IGD-C2** |  |  | 1.112 (.007)** |  |  | .973 (.031)* |  |  | .347 (.437) |
|  |  |  |  |  |  |  |  |  |  |
| ***F Statistic (df)*** | *.121 (3,54)* | *1.908(7,50)* | *2.589 (10,47)* | *.143(3,54)* | *.441 (7,50)* | *1.156 (10,47)* | *1.249 (3,54)* | *1.049 (7,50)* | *1.048 (10,47)* |
| ***R^2^*** | *.007* | *.211* | *.355* | *.008* | *.058* | *.197* | *.065* | *.128* | *.182* |
| ***Adjusted R^2^*** | *-.048* | *.100* | *.218* | *-.047* | *-.074* | *.027* | *.013* | *.006* | *.008* |
| ***p-value*** | *.947* | *.088* | *.014** | *.934* | *.872* | *.343* | *.301* | *.410* | *.420* |

**S1 Table. Relationship of memory scores, moderator variables and gender x memory interactions on model-free discounting measures.**

Each column represents one OLS regression. Dependent variables are shown in the column titles. Each row represents one predictor variable or statistics of the regression model. Cells show regression coefficients (beta) and p-values in brackets or model statistics.

* p < .05

** p < .01
