# Supplementary material for: Gender-Specific Differences in the Relationship between Autobiographical Memory and Intertemporal Choice in Older Adults
Source: PLoS One. 2015 Sep 3;10(9):e0137061. doi: 10.1371/journal.pone.0137061 (PMC4559386; doi:10.1371/journal.pone.0137061)
Supplement: S2 Table — Correlation coefficients and p-values (in brackets) are reported. All p-values are two-tailed. * p < .025. (DOCX) [file pone.0137061.s002.docx]

**S2 Table. Correlations of the different episodic memory tasks.**

|  |  | **IGD-C1** | **IGD-C2** |
| --- | --- | --- | --- |
| **FNPA-Performance** | **All participants** | .160 (.231) | .324 (.013)* |
|  | **Males** | .013 (.948) | .216 (.269) |
|  | **Females** | .132 (.488) | .157 (.409) |
| **IGD-C2** | **All participants** | .186 (.162) |  |
|  | **Males** | .387 (.042) |  |
|  | **Females** | -.009 (.964) |  |

Correlation coefficients and p-values (in brackets) are reported.

All p-values are two-tailed.

* p < .025
