# Supplementary material for: Gender-Specific Differences in the Relationship between Autobiographical Memory and Intertemporal Choice in Older Adults
Source: PLoS One. 2015 Sep 3;10(9):e0137061. doi: 10.1371/journal.pone.0137061 (PMC4559386; doi:10.1371/journal.pone.0137061)
Supplement: S1 Text — (DOCX) [file pone.0137061.s003.docx]

**S1 Text. Supplementary Information**

**Methods**

In addition to the intertemporal choice models mentioned in the main text, we used additional models to characterize our participants’ choices. To obtain a model free measure of discounting, the total number of choices for the smaller, sooner reward (not including the catch trials) made by each participant within one session was counted as the *number of impulsive choices (NImp)*.

Choice data of blocks 5 and 6 were used to calculate model-free measures of present-bias. Individual IPs of block 5 (*6 months vs. 9 months*) were divided by the IPs of block 1 (*tomorrow vs. 3 months*) and referred to as Present-Bias 3 months (PB-3). Similarly, the IPs of block 6 (*6 months vs. 12 months*) were divided by the IPs of block 2 (*tomorrow vs. 6 months*) and referred to as Present-Bias 6 months (PB-6). As the relative delay difference between the two rewards is the same in blocks 1 and 5 as well as in blocks 2 and 6, their ratios, PB-3 and PB-6, provide a measure of how much temporal proximity itself is valued.

**Results**

**Regression.** S1 Table shows the results of regression analyses using the three model-free discounting measures. Again three different models were analyzed. In line with the results regarding hyperbolic discounting parameter *k*, there was no significant effect of any of the memory scores on the overall discounting measure *NImp*, whereas the predictors *income* as well as the interaction between *gender* and *IGD-C2* were significant in further models.

In addition, results of the regression analyses with *PB-3* as dependent variable reflected findings with regard to present-bias parameter *β*. Although there was no significant contribution of *income*, we again observed a significant contribution of the interaction term *gender*IGD-C2* on *PB-3*. No measure significantly predicted *PB-6*.

**Correlations.** S2 Table shows correlations between scores of the different memory tasks. A significant positive correlation was found between FNPA-PF scores and IGD-C2 scores on group level, *r* = .324, *p* = .*013 < α = .025*, *r^2^* = 0.10, which is somewhat surprising considering the more semantic nature of the IGD-C2 task, but does suggest a relationship between these measures. Additionally, a correlation was found between IGD-C1 and IGD-C2 scores for men, *r* = .387, *p* = .*042 > α = .025*, *r^2^* = 0.15, however, this correlation was not significant after correction for multiple comparisons.
